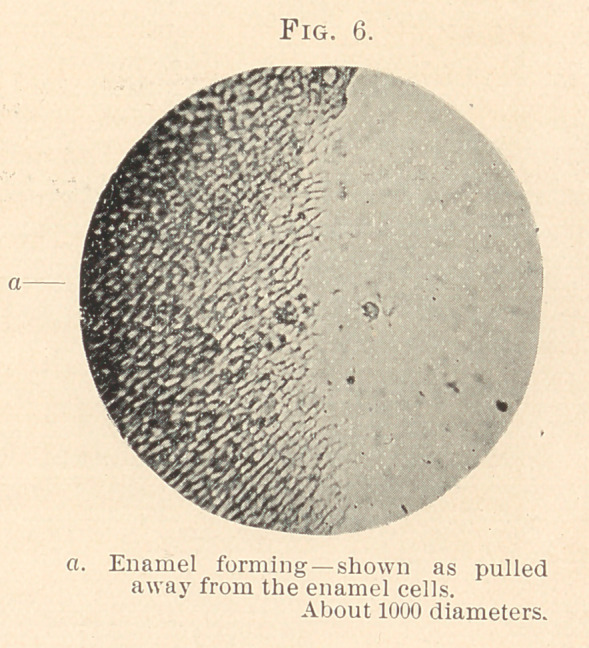# A Contribution to the Study of the Development of the Enamel

**Published:** 1893-12

**Authors:** R. R. Andrews

**Affiliations:** Cambridge, Mass.


### International Dental Journal.

Vol. XIV. December, 1893. No. 12.

### Original Communications.[1]

### A CONTRIBUTION TO THE STUDY OF THE DEVELOPMENT OF THE ENAMEL.[2]

BY R. R. ANDREWS, A.M., D.D.S., CAMBRIDGE, MASS.

In a paper presented before the Dental Section of the Tenth International Medical Congress at Berlin in 1890, I gave the result of my investigation on the formation of enamel up to that time. Since then my study leads me to believe that there exists in the young developing enamel something that has the appearance of fibres, guiding and sustaining the globules that are excreted from the enamel-cells which are to form the future enamel-rods, upholding and supporting them. An eminent English writer seems to imply that the study of dentine is the more difficult of the two, but my experience teaches me that the study of enamel development and calcification presents vastly greater difficulties. John and Charles Tomes were of the opinion that the enamel was formed by actual conversion of the enamel-cell; that the proximal end underwent some chemical change preparatory to its calcification, and subsequently calcified; that the calcification did not go on uniformly throughout the whole thickness, but from its outer surface towards

[1] The editor and publishers are not responsible for the views of authors of papers published in this department, nor for any claim to novelty, or otherwise, that may be made by them. No papers will be received for this department that have appeared in any other journal published in the country.

[2] Lecture delivered before the World's Columbian Dental Congress, Chicago, August, 1893.

its interior, the centre portion calcifying later than the external; that as this calcification proceeded it also united the contiguous cells to each other. Dr. J. L. Williams believes that the enamel-organ is glandular in its nature, and a true secreting organ. Appearances teach him that the ameloblasts are the active agents in depositing lime-salts on the periphery of the dentine. In teasing enamel-cells from partially formed enamel, he finds that they show a fibre running out from that end of the cell. He tells us that the enamel is probably formed, not by a change of the enamel-cells into a glue-yielding basis-substance, which afterwards becomes infiltrated with lime-salts, but by a process of secretion and deposition. As the formation of enamel progresses, these cells recede, leaving within the formed enamel what appears to be a fibre of living matter in the centre of the enamel-prism.

Professor W. X. Sudduth, in an article in the "American System of Dentistry," is of the opinion that during the period of amelification there is no conversion of living tissue into enamel, but that the enamel is produced by a process of excretion. He finds that at first the salts of calcium are stored up in the meshes of the stellate reticulum of the enamel-organ, furnishing material for the first-formed layer of enamel. After this, the enamel-organ having disappeared from over this calcified layer, the salts of calcium are furnished by a rich plexus of capillary vessels, which are now found to be in direct communication with the enamel-cells. He believes that the fibrils, which have been called "Tomes's processes," are nothing more than semi-calcified material that adheres to the enamel-cells, giving the appearance of a fibril or a prolongation of the cells themselves. He considers them as being mechanically made, for they do not always appear, but depend upon a certain condition of the calcific material. He had succeeded in demonstrating them in the enamel-cells of pigs' teeth, where they showed very plainly, indeed, being nearly or quite as long as the cells themselves, and several times longer than the enamel was thick. As a rule, he finds that the enamel-cells separate from the forming enamel so as to leave a comparatively smooth line. He has never been able to demonstrate processes that would lead him to infer the least analogy between them and the fibrilla of odontoblasts. He seems at a loss how to regard the cells of the stratum intermedium, and says, "Just what their significance is I am unable to state positively. I am led to believe that they supply the places made by the increase in the circumference of the enamel, and account for the short prisms seen in ground sections of the enamel. Their office is to develop ameloblasts to supply the places of those which are carried up with the growing tooth." To him the enamel is simply a coat of mail supplied by nature to protect the dentine.

Kölliker believes that the process of calcification is one of secretion. The "Tomes processes" he considered as being fragments of the hardened secretion, which are still clinging to the parent cell. Schwann believed that the enamel-cell was constantly increasing at the end next the enamel, and that the new growth or younger part is calcified as soon as it is formed. E. Klein says that the enamel is formed by the enamel-cells in the same manner as the dentine from the odontoblasts,—that is, the distal extremity of the cells next the dentine elongates, and this increment is directly converted into enamel.

I wish to repeat what I have already said before, that the continual sheet of tissue that can be raised from young developing enamel is not a membrane, and I think most of the more recent authorities agree with me. Charles Tomes gives us to understand that it is something produced solely by the destructive action of acids; but this I am quite sure will be found to be a mistake. Mr. Mummery has shown that this layer exists in teeth which have not been subjected to the use of acids. It is only that part of the enamel first formed that is not wholly calcified. The enamel cells that have been properly prepared and not shrunken will be seen filled with minute globules, highly refractive, and supplied, when the enamel is first formed, from the meshes of the stellate reticulum rich in lime-salts at this time.

When the stellate reticulum is absorbed, as it is just after calcification commences, the lime is supplied by a rich capillary net-work in contact with the enamel-cells. The authorities who speak of granules of lime have described them as seen in the shrunken cells in the tissue as it is usually prepared. They are really globular, though minute. If, just as calcification commences, we place a few drops of dilute nitric acid on the slide near the edge of the cover glass, it will, by capillary attraction, run under, and these refractive granular bodies in the stellate reticulum will disappear, as will those that are in the enamel-cells themselves. Large numbers of small bubbles will accumulate, and force themselves out from under the cover glass. This would seem a positive demonstration of the presence in the stellate reticulum and enamel-cells of carbonate of lime just previous to commencing calcification.

I am convinced that these minute, refractive bodies are calcospherites, that are taken in by the active enamel-cells, and excreted from them where, by coalescing they form larger globules, and these form the rods. You will pardon me here if I quote briefly from my former paper on this subject. In teasing off portions of active enamel-cells from enamel forming, I have found the surface of the dentine on which it is being formed covered with layers of globules that have been deposited there by the enamel-cells. These given out from the cell continually form, by coalescing, larger globules, and these become the enamel-rods. One rod is separated from another by what appears to be a protoplasmic substance. This substance in many of my sections projected out beyond the line of calcification, and appeared as though it was a process or a fibre; of this I shall speak farther on.

Many of my sections of forming enamel were purposely cut extremely thin, that I might study a single thickness of the layer of the cells, and these specimens were not stained; therefore some of them do not show as clearly in the photo-micrographs as I could wish. Yet I think I shall be able to demonstrate the points of which I speak. Others of these photographs will illustrate it almost as a diagram. Some little time after finishing my former paper on "Enamel, its Development and Calcification," I read it to Professor E. L. Mark, of the Biological Department of Harvard University. He stated that I had found and demonstrated new points about the enamel that a German investigator had recently described; that both papers gave similar views. At my request he translated the paper from the German journal in which it was published. Its title was "On the First Processes of the Deposition of the Enamel," by Dr. Graf Spee. The description he gave of this process was so nearly like my own, that I read it with considerable surprise.

I did not know that any one had described the enamel-rod as being formed by minute globules coming through the cell. But he had seen these minute and highly refractive globules in the body of the cell, and says that when the tissue is properly prepared—and he lays great stress on this point—they are always to be found there at the time of the formation of the enamel. Their entire absence at earlier stages is an indication that these globules are an enamel substance. He gives to them the name "enamel-drops," and says he saw these "enamel-drops," when enamel is to be formed, appear only in the half of the enamel-cells which rests on the dentine; afterwards farther up in the cell, but not quite up to the region of its nucleus. Many of them were so small as to be scarcely measureable, and they are always spherical. Great numbers of them are collected at the periphery, and appear here either to be completely merged or fused together. The lower part of the cell contains the larger "enamel-drops," which merge without sharp boundaries into the substance of the enamel-rods. This then appears as a part of the enamel-cell, in which the originally isolated "enamel-drops" have run together into a continuous mass, and the growth of the enamel rod once begun, appears to take place by the addition of new "enamel-drops." Dr. Spee's "enamel-drops" were really what I described as minute calco-spherites, which, merging together, had formed larger globules, of a substance which I believe to be calco-globulin.

Perhaps the most important contribution to the literature of dental histology during the last decade is a paper entitled, "Some Points in the Structure and Development of Dentine," by J. Howard Mummery, M.R.C.S., L.D.S., of London. This paper was read before the Royal Society, March 5, 1891. Appearances noted and demonstrated by Mr. Mummery in this paper recalled to my mind similar appearances which I had seen in the developing enamel, appearances that I could not then explain, that I did not understand. As some of his results will be of interest to us while considering my subject, I shall try and give a brief idea of them. He noted the appearance of connective-tissue fibres or bundles of fibres in advance of the main line of calcification, whose high refractive index suggested their partial calcification, these processes being continuous from the formed dentine to the general connective tissue of the pulp. He found in a young developing tooth a distinct reticulum of fine fibres passing between and enveloping the odontoblasts. By a careful focusing, he saw these fibres gathered into bundles and incorporated with the matrix-substance of the dentine, out of which they seemed to spring. The origin of these fibres would seem to be from connective-tissue cells which are found everywhere in the pulp next the odontoblastic layer, and also, as he demonstrates, between the odontoblasts themselves.

He continues: "We can no longer look upon the matrix of dentine as being a homogeneous substance, but must regard it as composed of a net-work of fine fibres of connective tissue, modified by calcification, and, where that process is complete, entirely hidden by densely-deposited lime-salts." His investigation as to the occurrence of this tissue suggests this view, that these fibres are the scaffolding on which the tooth-matrix is built up; that they are incorporated in the matrix of the dentine, and form really the basis of its substance. Mr. Mummery's article is convincing and admirably demonstrated. His investigation was carried on after the process of Dr. L. A. Weil, of Munich, which consists of first fixing the soft parts of developing tooth in a saturated solution of corrosive sublimate in water. When fixed, the sublimate is removed by washing, and the specimen pressed through successive strengths of alcohol to absolute alcohol, then into chloroform, to which are gradually added fragments of desiccated Canada balsam, until a very thick solution of the balsam is produced. It is then allowed to penetrate and become hardened by keeping in a warm temperature. After the balsam is hard the specimen is removed, cut with a fine, sharp saw under water, as there has been no decalcification, and the sections thus cut are ground down first on a lathe with corundum and afterwards on a fine stone with water under the finger. Sections are then mounted in Canada balsam.

I have tried this process without any success on the developing enamel. The tissue at this time is too delicate to stand this treatment, and results in my hands have been failures—perhaps from a want of more practice in this method. But I do not believe it can be used with success when the tissues are so delicate. Certainly here the investigation of enamel is more difficult than that of dentine. The method is admirable for investigating fully-formed structures, as it shows the organic tissues in undisturbed relation to the calcified tissues. Appearances of calcified fibres projecting beyond the line of calcification I have already seen in young forming enamel, and I commenced a series of investigations to see if I could find out what these appearances indicated.

I commenced by trying to tease apart enamel-cells; after some little experimenting I am quite sure I found evidence that processes from the cells of the stratum intermedium of the enamel-organ pass down through and among the ameloblasts to the forming enamel beneath. (See Fig. 1.) And I judge that these are the processes which Mr. Tomes saw and described as processes connecting the enamel-cells with the cells of the stratum intermedium. I then commenced a series of experiments, trying to separate slightly the layer of enamel-cells from the stratum intermedium. The parted edges had the appearance of broken processes, and in several specimens there are processes crossing from the enamel-cells to the stratum intermedium. I shall try to demonstrate this appearance to you, although I confess I have a difficult task; the teased and pressed tissue and the different depths of the tissue make the matter a difficult one. I think I shall be able to indicate what I mean by my photo-micrographs. (See Fig. 2.)

A longitudinal section of a human tooth at birth, just after the process of calcification in the enamel has commenced, will show, between the enamel-cells and the formed enamel, a thin layer which has been called by earlier investigators the membrana performativa. (See Fig. 3, *b*.) It was misunderstood then ; it is not a membrane. It is the latest deposition of enamel from the enamel-cells, composed of globules or masses of calco-globulin ; and around these globules there seems to be a fibrous net-work. Connecting with this fibrous net-work and running to the formed enamel beneath, we find innumerable thread-like processes, appearing like fibres. (Fig. 3.)

In several of my specimens this shows with great distinctness. (See Fig. 4, *a*.) There are indications of fibres which have been broken on the upper portion of this thin layer which appear as though they had been broken off in the separation of the layer from the enamel cells. In a longitudinal section of the tooth of a calf at birth, where the recently-formed layer of enamel is still in contact with the fully-calcified enamel, I have succeeded in teasing off this younger portion and exposing to view what appear to be fibrils standing out from the surface. (See Fig. 5.) These have apparently been drawn out from the only partially calcified new tissue.

In another longitudinal section from calf at birth are to be seen, on that part of the enamel broken away from the enamel-cells, processes standing out like so many coarse threads. (See Fig. 6.) They appear so large that it is probable they have been enlarged either by the action of reagents or by calcific matter clinging to a fibre, if one is there; and they are undoubtedly partially calcified. They are very much coarser than the fine fibrils which I saw between the enamel-cells. Deeper within, these processes are seen to surround the globules or masses which have been deposited by the enamel-cells, and which are forming the rods.

In another section from tooth of calf, the younger layer of forming enamel shows a net-work of fibres, of which I have already spoken. They are surrounding the recent deposition of globules. It is only in this layer that I have been able to demonstrate this appearance. I have not been able to see this net-work in more fully-formed enamel, but a distinct net-work is always visible in that layer first deposited.

In regard to the processes of the cell, Tomes first describes them, and says they are due to the manner in which the cell calcifies. In his illustrations they are shown as coming from the base of the cell, from the centre, and from the extreme edge. Sudduth believes that they have nothing whatever to do with the cell, as being mechanically made. He pictures them as coming from the base of the cell, from both sides of it, and from between the cells.

Williams, as I have already quoted, states, "As the formation of enamel progresses, these (enamel) cells recede, leaving within the formed enamel what appears to be a fibre of living matter." We have here, you see, a variety of opinions from excellent observers. My investigations lead me to believe that these processes may have their origin among the cells of the stratum intermedium; that they pass either within or between the enamel-cells, and thus on, to form a fibrous sub-structure, among which are deposited the globules which are to form the future enamel rods.

When the calcification of the rod is complete, the lime-salts have been so densely deposited as to entirely obscure the appearance of any fibre. The stratum intermedium, in which, as I have stated, I have reason to believe these processes originate, has been thought by more than one observer to be a species of connective tissue. Of this fact I am not certain. But it is certain that, after calcification commences, the connective tissue of the jaw is in direct contact with the cells of the stratum intermedium, and the cells from this stratum must be and are recruited from the connective tissue of the parts.

Tomes, who has done much in describing human and comparative dental anatomy, has shown us that fibrils exist in the enamel of the kangaroo and in the Sargus, or sheep's-head fish. In this fish the enamel is penetrated by a system of what he describes as tubes, which are not continued out of, or derived from, the dentine, but belong to the enamel itself. They give off numerous branches. This peculiar appearance led Kölliker to believe it was not true enamel, but Tomes proves that, being developed from an enamel-organ homologous with and exactly like that of amphibia and reptiles, the tissue must certainly be regarded as unquestionably enamel.

To sum up my conclusions: I am led to believe that there probably exists in developing enamel, as has already been found in developing bone and dentine, a fibrous sub-structure on and between which the enamel is deposited. After the enamel is wholly formed, its existence seems to be wholly blotted out in the dense calcification of the tissue. In sections of wholly-formed enamel I have never been able to trace it, although I have tried the methods of those who claim to have seen it. In regard to the beaded protoplasmic reticulum of living matter in formed enamel, I have never been able to find it. I believe with Klein, that it is improbable that nucleated protoplasmic masses are contained in the interstitial substance of the enamel of a fully-formed tooth. I wish, in closing, to acknowledge my indebtedness to Professor George A. Bates, of the Boston Dental School, for the use of specimens of sections of human teeth at the time of birth, which he had prepared, and from which several of my best photo-micrographs have been made.

## Figures and Tables

**Fig. 1. f1:**
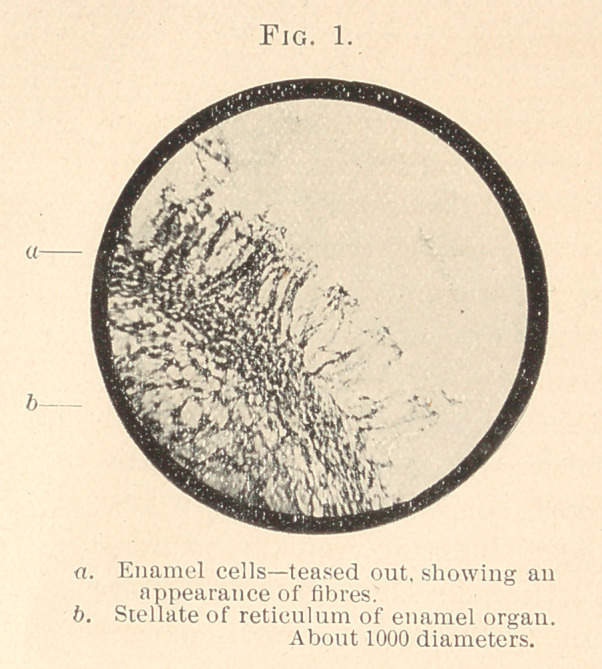


**Fig. 2. f2:**
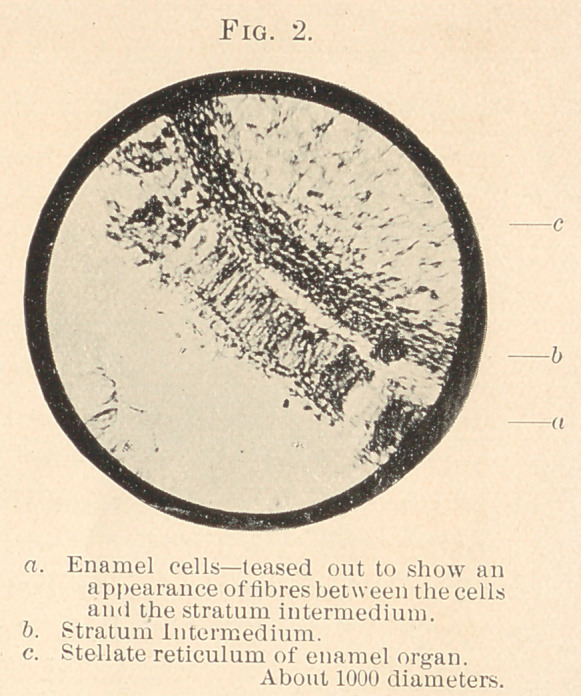


**Fig. 3. f3:**
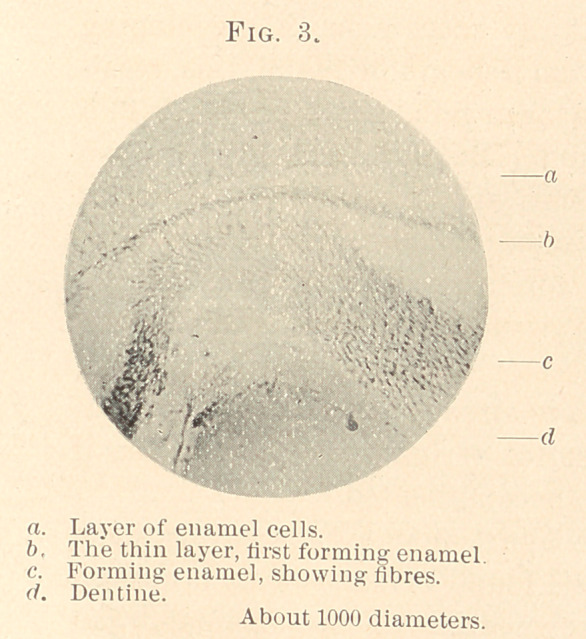


**Fig. 4. f4:**
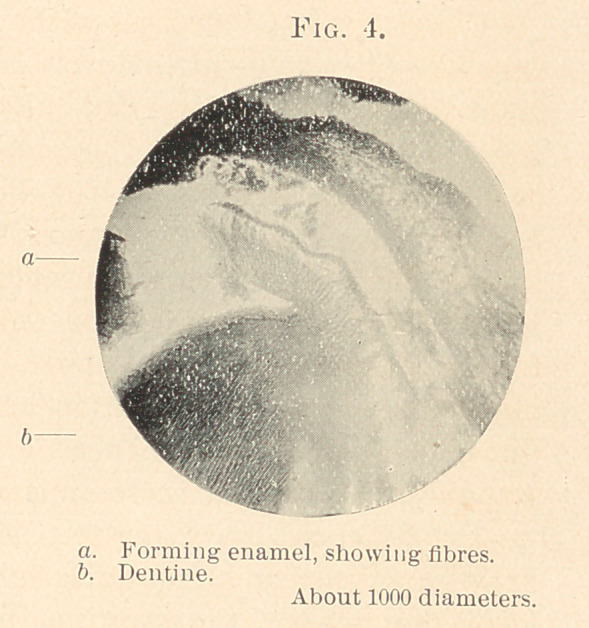


**Fig. 5. f5:**
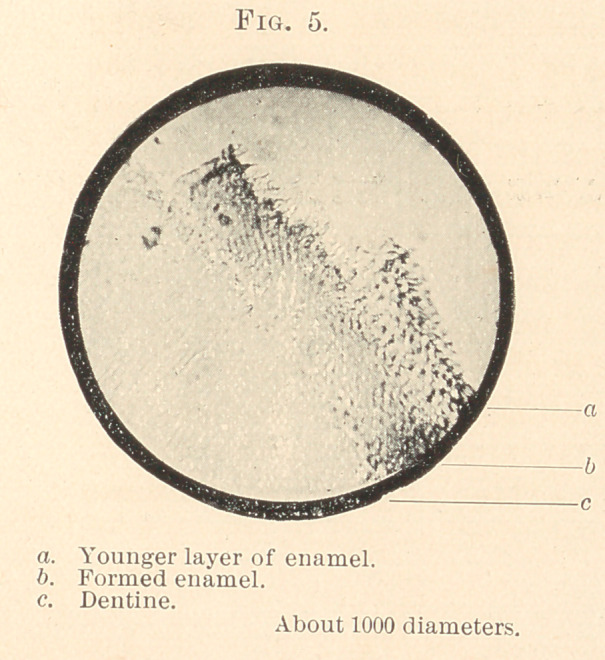


**Fig. 6. f6:**